# Evaluation of a smartphone food diary application using objectively measured energy expenditure

**DOI:** 10.1186/s12966-017-0488-9

**Published:** 2017-03-14

**Authors:** Felicity J. Pendergast, Nicola D. Ridgers, Anthony Worsley, Sarah A. McNaughton

**Affiliations:** Institute for Physical Activity and Nutrition (IPAN), School of Exercise and Nutrition Sciences, Deakin University, 221 Burwood Highway, Burwood, Victoria 3125 Australia

**Keywords:** Dietary assessment, Smartphone applications, Evaluation, SenseWear armband, Energy intake

## Abstract

**Background:**

Dietary assessment methods are limited in their ability to adequately measure food and beverage consumption. Smartphone applications may provide a novel method of dietary assessment to capture real-time food intake and the contextual factors surrounding eating occasions. The aim of this study is to evaluate the capability of a Smartphone meal diary app (“FoodNow”) to measure food intake using a validated objective method for assessing energy expenditure among young adults.

**Methods:**

Participants (18–30 years) used FoodNow over four non-consecutive days recording all eating occasions through a combination of written text, and/or optional images and voice recordings. A series of contextual questions were also completed. Participants wore the validated SenseWear Armband (BodyMedia Inc, USA) during the same period to measure free-living energy expenditure. Intra-class correlation coefficients (ICC) estimated the reliability of FoodNow to measure estimated energy intake compared to measured energy expenditure.

**Results:**

Ninety participants (71 female, 19 male; mean age = 24.9 ± 4.1 years) were recruited to use the FoodNow app to record their eating occasions. Thirteen were excluded as they did not meet minimum requirements for number of reporting days (*n* = 3) or SenseWear Armband wear time (5 days of 11 h), while 21 participants were excluded after being identified as mis-reporters (Huang method). Among the remaining sample (*n* = 56), reliability between estimated energy intake and measured energy expenditure was high (ICC, 95% CI: 0.75, 0.61–0.84).

**Conclusions:**

FoodNow is a suitable method for capturing estimated energy intake data from young adults. Despite wide levels of agreement at the individual level (−3709 kJ to 2056 kJ), at the group level, FoodNow appears to have potential as a dietary assessment tool. This new dietary assessment method will offer an alternative and novel method of dietary assessment which is capable of collecting both estimated energy intake and contextual factors surrounding eating occasions. Information collected may be used to inform future public health messages or research interventions.

## Background

The development of accurate dietary assessment methods has been the focus of many research ventures within nutritional epidemiology [1]. Commonly used methods for assessing food intake are food diaries, 24-h recalls, and food frequency questionnaires [2]. Food frequency questionnaires have been used widely due to their low respondent burden, however they are unable to assess a number of important dietary exposures relating to eating patterns and the eating context [3]. Only 24-h recall methods or food diaries or records can provide the necessary data to examine eating occasions, however 24-h recall methods depend on episodic memory processes and are subject to recall biases [3].

Due to the inherent complexities in assessing what people eat, the field of dietary assessment has looked to technology to assist in measuring food intakes [4]. The use of technology has aimed to decrease subject burden, improve reporting by reducing measurement error and bias, and finally decrease researcher burden by decreasing costs and resources associated with data collection, coding and reporting [4]. To date, applying technology in dietary assessment has tended to focus on introducing improvements relating to data entry and mode of administration (e.g. mobile and web-based tools) [5], improvements relating to coding and analysing food intake [6] and augmentation of data collection (e.g. use of wearable devices/cameras) [4, 7].

The application of technology to food diaries has focused on adapting them for use with personal digital assistants and Smartphones [6, 8–10], incorporating the use of images (with or without automated coding of intake) [11–13], push notifications [6], voice recording options [14] and geo-coding via GPS location information [5]. These developments have the potential to reduce researcher and participant burden, improve data quality, increase adherence, and provide real-time data collection and communication, whilst reducing the need for manual dietary coding [4, 5]. Furthermore, this technology may reduce the total costs associated with dietary assessment and have the potential to increase the scope of contextual information collected about eating occasions [5].

Smartphone technology, given its widespread uptake and pervasiveness, has provided new opportunities for nutrition research including dietary management, intervention tools [15, 16] and the assessment of dietary intake, specifically through the development of electronic food diaries. A range of developments in electronic food diaries have been implemented which vary in complexity, whether images are involved and whether coding is automated. For example, some electronic food diaries are primarily adaptations of paper-based diaries allowing allow text entry of food intake that require subsequent coding by trained staff [9, 17]. Some food diaries, particularly commercially available apps such as Australian Calorie Counter – Easy Diet Diary [18], MyFitness-Pal [19], and Im2cal [20] are directly linked to food compositions database so that no coding by study personal is required. Other developments include the use of images to augment food descriptions [11, 13] or to allow automated coding by volume estimation [6]. However, more research is required to validate the use of Smartphone food diaries for assessing dietary intake.

Assessing the validity of dietary assessment methods involves comparing the new or test method with another method, usually referred to as the reference method. Existing electronic food diaries have been evaluated using a variety of methods as the reference method including doubly-labelled water (DLW) [6, 21, 22], 24-h recalls [8, 23, 24], weighed food records [10, 25] and estimated food records [26, 27]. Each method has limitations; DLW because of its high cost, invasiveness, specialised equipment for analysis and the need for trained personnel [1]. 24-h recall’s rely strongly on participant recall and therefore have human memory bias limitations [2], while weighed and estimated food records are limited due to their high participant and researcher burden and misreporting [1]. Recently, however, the SenseWear Armband (SWA) has been shown to be an accurate, reliable and less burdensome method compared to DLW for measuring energy expenditure (MEE) [28, 29]. The SWA is a small multi-sensor lightweight device worn on the upper arm, which has been validated within numerous populations, health conditions and exercise intensities [28, 29]. This method provides a feasible, valid tool for the assessment of free-living energy expenditure for validating dietary assessment methods. Given the importance of evaluating newly developed dietary assessment tools, the aim of this study is to evaluate a Smartphone meal diary app which measures food intake using a validated objective method for assessing EE among young adults.

## Methods

### Procedure

This cross-sectional study was conducted among 90 young adults aged 18–30 years. Recruitment was conducted between June and December 2014 and restricted to participants living in the state of Victoria, Australia. Women currently pregnant or lactating were excluded from recruitment due to potential variations in their eating habits and food intake. Sample size was determined based on recommendations and previous studies of validation of dietary intakes methods [30].

Recruitment methods included both online (Facebook, Twitter) and physical methods (poster advertisements, flyer distributions). Participants were required to attend the research clinic at Deakin University (Burwood campus) on two occasions. At the first visit, informed written consent was obtained, a self-administered questionnaire was completed, and anthropometric measurements were taken. During this visit, the participants were provided with both the FoodNow app, a fiducial marker card (provides scale for measurements of other objects in images), and the SWA (Model MF-SW, BodyMedia, Pittsburgh, USA). At the second visit (approximately 1 week after the first visit), the equipment was returned, body weight measured and a thank you letter containing a $25 voucher (compensation) was given to each participant.

Ethical approval was received from Deakin University Human Ethics Advisory Group, Faculty of Health (HEAG-H) in April 2013 (Ethics Number HEAG-H 31_2013).

### Measures

#### Eating patterns and energy intake

The FoodNow meal diary app was developed to address an existing gap in the measurement of dietary behaviours and has not been reported on previously. The FoodNow app allowed the participants to record all foods and beverages consumed as an eating occasion and was designed to be downloaded onto the participants own phone. Both IPhone and Android platforms (the two largest Smartphone providers) were compatible with the FoodNow app. The FoodNow app contained three features to facilitate recording of food and beverage intake at each eating occasion, that is, the ability to capture an image of the eating occasion, record a text description of the eating occasion and the ability to record a voice message describing the food and beverages consumed. Only the text description was compulsory to collect.

The following provides a step-by-step description of the process of recording an eating occasion, in the order requested by the app. Firstly, prior to the consumption of an eating occasion participants were encouraged to take two images of the food items, one taken directly above the food item, the second from a 45° angle. All food and beverage items and the fiducial marker needed to feature in all images. The fiducial marker was a standardized business card provided to the participant, which gave a size reference for each image [31]. These image-based methods were based on approaches used in previous studies [5, 31]. If it was not possible to take an image, participants could bypass this step by selecting “No image”.

Subsequently, participants were asked to provide a text description of the foods and beverages they were about to consume. The text description was mandatory for each eating occasion and requested information on the food items, including the type of food and brand name, as well as amounts of each food item. Finally, participants were given the opportunity to provide a voice recording of any additional information of the food items or amounts that were consumed. The collection of images and voice recordings were not mandatory yet highly encouraged by research staff.

Post consumption, the participant completed a question relating to food wastage and took an image of any remaining food items. This was to account for over-reporting of food consumption [4]. Again, if it was not possible to take an image, participants could bypass this step by selecting “No image”. Push notifications were sent to participants if they failed to report anything in the app for a 3 h period during waking hours (9 AM–9 PM), and acted as reminders/prompts to use FoodNow.

Finally, after recording the food and beverage intake at the eating occasion, the participant was prompted to complete questions in FoodNow relating to contextual factors of eating occasions (e.g. where they ate, who they ate with, what they were doing when eating, who prepared and purchased the food items, cooking methods and meal preparation time) [32]. The day following a reporting day, participants were required to complete questions regarding sleep times, dietary supplement consumption, and whether the previous day reflected usual consumption. Prior to evaluation, FoodNow was extensively pilot tested in-house by staff and students from the Institute for Physical Activity and Nutrition Research (IPAN), Deakin University.

Participants were asked to record their eating occasions in FoodNow on four non-consecutive days (3 weekdays and 1 weekend day) over a period of 1 week. Previous research has highlighted the need for both weekday and weekend collection days to control for day-to-day variation and occasionally consumed foods [1]. Non-consecutive days were used to control for misreporting errors related to consecutive day data collection [33]. Four data collection days were chosen as a compromise between capturing day-to-day variation and the practicability of participant burden [1].

Coding of the dietary data involved matching each food/beverage item from the text description in the FoodNow app to an appropriate item based on nutrient composition and quantity, using the 2011–2013 Australian Food and Nutrient Database [34]. Images, voice recordings and contextual question responses (cooking method) were used to increase the coding accuracy by complimenting the text description of the food/beverage items and amounts. A fiducial marker was used as a size reference when assessing the images to assist with coding the amount of food consumed. Coding of the data was completed by three nutritionists who were fully trained in this coding technique. Data was checked for accuracy through a duplicate nutritionist review process, which involved double checking each food code and amount. If a consensus was not reached between the two coders, a dietitian was consulted and a consensus approach was used. Total estimated energy intake (EEI) per participant per day was calculated by summing the energy content of all food and beverage items consumed and averaged over the number of reporting days. Participants’ were excluded if they had less than three days reporting days, which has been previously reported to be sufficient for dietary assessment [1].

### Energy expenditure

Measured energy expenditure (MEE) was captured by the SWA. The SWA is a multi-sensor monitor (tri-axial accelerometer, heat flux, galvanic skin response, skin and near-body ambient temperature sensors) that is worn on the left upper arm over the triceps muscle and is validated for use within numerous populations, health conditions and exercise intensities [28, 29]. Physiological data were sampled at 32 Hz. Each monitor was configured for participants using proprietary software (SenseWear Professional v7, BodyMedia Inc). Participants were required to wear the monitor for seven days excluding water-based activities. This period controls for day-to-day variability in exercise and MEE, [28], and has been validated for a period of seven days in previous studies [29]. At the conclusion of data collection, SWA data were downloaded and processed in 1-min epochs using algorithms within the proprietary software.

To date, no studies have reported how many hours/day or the number of days that are required to reliably determine free-living total EE in young adults using the SWA. Wear time criteria were calculated for total EE using the Spearman-Brown prophecy formula [35]. To achieve a reliability of ≥0.9, 4.5 days of at least 11 h/day wear time was required, which was rounded up to the next whole day to ensure that the reliability criteria was met. Consequently, five valid days of 11 h wear time was set as the inclusion criterion for this study.

### Anthropometry

Participants’ height, body weight and waist circumference was measured using standardised protocols [36]. Body mass index (BMI) (weight (kg)/height (m)^2^) was calculated from these measures and used to categorise participants into underweight (BMI <18.5) normal range (BMI 18.50–24.99), overweight (BMI ≥25.00) and obese (BMI ≥30.00) as defined by the World Health Organization [37]. Body weight was recorded again at visit 2. This second weight measurement was taken to control for potential weight change over the week long study period.

### Covariates

Covariates including age, sex, special diet, smoking status, country of birth, highest qualification and postcode were collected using a self-reported questionnaire at visit 1. Special diet was defined as actively trying to lose weight. Post code was used to assign participants via the Australian Bureau of Statistics Socio-economic Index for Areas (SEIFA) into a low, medium or high socio-economic category ranked by their suburban postcode [38].

### Data analysis

Data analysis were conducted using Stata Statistical Software, v14 (Stata Corporation). Descriptive statistics were used to report anthropometric and demographic characteristics. Pearson correlation coefficients were used to assess the strength of the association between EEI and MEE. The ICC of absolute agreement was also calculated to evaluate the relationship between EEI and MEE. The ICC accounts for both the difference between measures and the degree of correlation [39]. Both methods are commonly used in the evaluation of dietary assessment methods [1]. A Bland-Altman plot was used to analyse the limits of agreement between the two variables by calculating the standard deviation of the difference between the two measures [40]. A p value <0.05 was considered statistically significant.

The level of misreporting observed was also assessed. This was based on the fundamental principle that EE = EI ± body stores, where, in the absence of a non-significant weight change (stable weight) at the group level, the expected ratio of EE: EI is 1 [41]. Inaccurate reporting of dietary EI is now widely recognised, and has been observed to vary from 10 to 50% [42]. Misreporting, characterized by the reporting of implausible EIs may undermine the validity of the method. The participants were classified as adequate-reporters (AR), under- reporters (UR) or over-reporters (OR) of EI in accordance with the Huang method [43]. This is an updated method for identifying physiologically implausible dietary reports by comparing EEI with MEE according to whether the individual’s ratio was within, below or above the 95% confidence intervals of the expected ratio of 1.0. It is able to account for intra-individual variation in EI by using the CV_EEI_ over the number of days (d) of intake data. This method resulted in 95% CI of <0.73 and >1.27, any participant outside of these cut offs were excluded from analysis.

## Results

### Analytical sample

Ninety young adults (71 female, 19 male) aged 18–30 years (mean age: 24.9 ± 4.1 years) from the Melbourne region, Victoria, Australia were recruited for this study. The majority of participants, were within the healthy weight range (BMI 18.5–24.9), non-smokers, born in Australia, lived in a suburb ranked with a high SEIFA score and held university degrees (Table 1).

**Table 1:** Characteristics of the young adult participants (*n* = 90)

Characteristic	
Age (years), Mean ± SD	24.9 ± 4.1
Sex (female), n (%)	71 (79)
Height (m), Mean ± SD	1.7 ± 9.2
Weight visit 1 (kg), Mean ± SD	65.7 ± 13.0
Weight visit 2 (kg), Mean ± SD	65.4 ± 13.3
Body mass index (kg/m2), Mean ± SD	22.84 ± 3.1
Weight status, n (%)	
Underweight (BMI < 18.5)	3 (3)
Healthy weight (BMI 18.5–24.9)	68 (76)
Overweight (BMI 25–29.9)	17 (19)
Obese (BMI > 30)	2 (2)
Special diet (no), n (%)	89 (99)
Smoker (no), n (%)	88 (98)
Country of birth, n (%)	
Australia	76 (84)
Other	14 (16)
SEIFA, n (%)	
Low	4 (4)
Medium	17 (19)
High	69 (77)
Highest qualification, n (%)	
Higher school certificate (Year 12 or equivalent)	16 (18)
Trade/apprenticeship (e.g. hairdresser, chef)	3 (3)
Certificate/diploma (e.g. childcare, technical)	8 (9)
University degree	43 (48)
Higher University degree (e.g. Graduate Diploma, Masters)	20 (22)

*SEIFA* Australian Bureau of Statistics Socio-economic Index for Areas

**Fig. 1:**
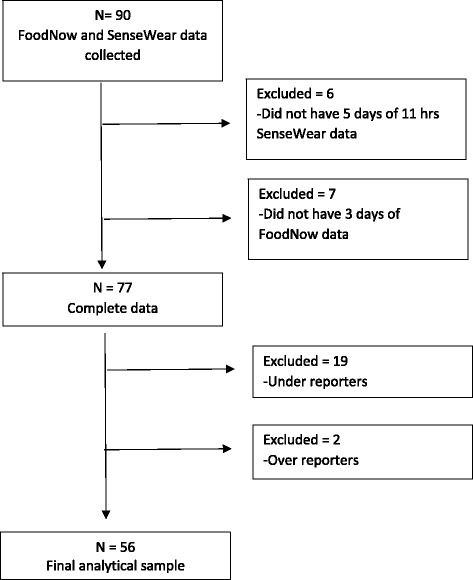
STROBE-nut flow diagram of analytical sample

The mean weight of participants was not significantly different between visit one and visit two (*p* = 0.9075). Participants with less than three days of dietary data (*n* = 7) and who did not meet the wear time criterion for the SWA (*n* = 6) were excluded from further analysis. Analysis of misreporting identified 19 participants as UR, two participants as OR, resulting in 56 AR (Fig. 1). There were no significant differences (*P* < 0.05) between those classified as UR, AR, OR, or those excluded on the basis of inadequate days of reporting for either dietary intake or SWA (Table 2). Further analysis of agreement was restricted to AR only (*n* = 56).

**Table 2:** Differences in under reporters, adequate reporters, over reporters and those who were excluded from analysis

Characteristic	Under reporters (*n* = 19)	Adequate reporters (*n* = 56)	Over reporters (*n* = 2)	Excluded (*n* = 13)
Age (years), Mean ± SD	23.90 ± 2.61	25.1 ± 4.68	24.97 ± 1.82	26.00 ± 2.78
Sex (female), n (%)	14 (74)	41 (73)	2 (100)	12 (92)
Body mass index (kg/m^2^), Mean ± SD	23.53 ± 2.24,	22.79 ± 3.16	22.28 ± 1.94	22.16 ± 3.56
Country of birth (Aus), n (%)	16 (84)	48 (86)	2 (100)	10 (77)
SEIFA (high), n (%)	16 (84)	44 (79)	1 (50)	8 (62)
Highest qualification (university degree or higher), n (%)	14(74)	39 (70)	1 (50)	9 (69)
Smoking Status (smokers), n (%)	0	1 (2)	0	1 (8)

*SEIFA* Australian Bureau of Statistics Socio-economic Index for Areas

Among the analytical sample (*n* = 56), every participant recorded at least one image over their reporting period with 14% of participants providing images for every eating occasion. Overall, 72% of 1339 eating occasions reported by the analytical sample had images that were used in dietary assessment coding. At the participant level, among those missing one or more images (*n* = 48), the number of eating occasions without images (mean ± SD per participant) was 7.9 ± 6.4 or approximately 33%.

### Measures of agreement

**Fig. 2:**
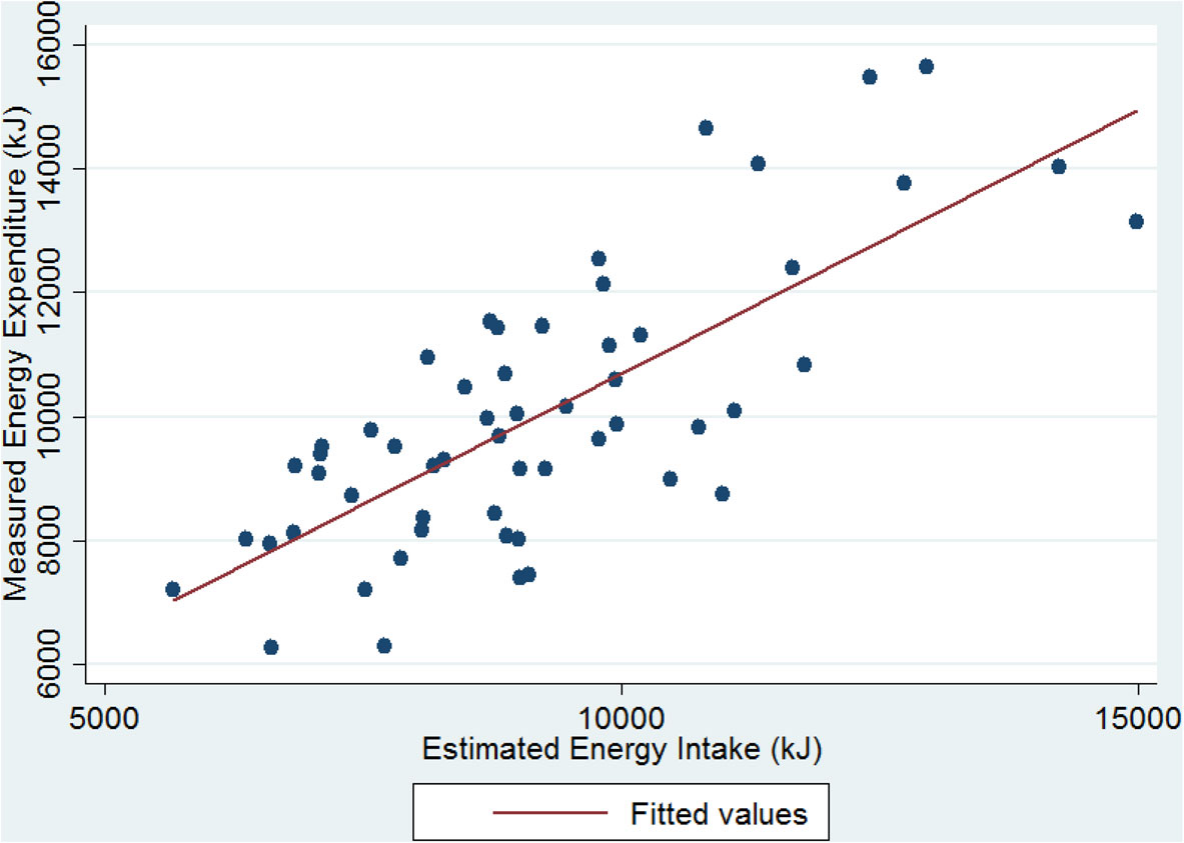
Scatterplot between measured energy intake and estimated energy intake with regression line (*n* = 56). kJ: Kilojoule

The mean (±SD) EEI recorded by the FoodNow app was 9204.14 kJ (1957.61) compared to the MEE measured by the SWA which was 10030.43 kJ (2210.35). Figure 2 is a scatter plot between MEE and EEI including a regression line to indicate agreement between the two methods.

Correlation coefficients (two-way random-effects model) between methods revealed that the relationship between EEI measured by FoodNow and MEE obtained by the SWA was strong. A high degree of reliability was found between EEI and MEE (ICC, 95% CI: 0.75, 0.61, 0.84) (*p* > 0.0001). There was a positive Pearson’s correlation between EEI and MEE (*r* = 0.75, *p* > 0.0001, *R* ^2^ = 0.56).

**Fig. 3:**
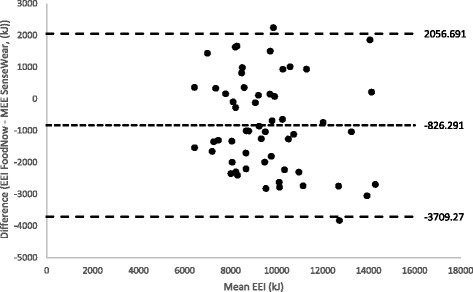
Bland-Altman plots between the mean estimated energy intake (EEI) and the difference in EEI and MEE in 56 young adults (*n* = 56). EEI: Estimated energy intake, MEE: Measured energy expenditure

Bland-Altman plots were used to illustrate the agreement between EEI measured by the FoodNow app and the MEE measured by the SWA (Fig. 3). The mean difference between EEI and MEE was 826.29 kJ, but the 95% limits of agreement were wide (95% CI: −3709.27, 2056.69) indicating error at an individual level. No systematic bias was detected, with random scatter of data points seen in Fig. 3.

## Discussion

This study evaluated an innovative method of dietary assessment using a Smartphone app “FoodNow” within young adults. The results show that FoodNow captured a mean EEI that was strongly correlated to the MEE measured by the SWA in young adults aged 18–30 years. FoodNow as an EEI measurement tool is comparable to previous Smartphone dietary assessment approaches [8, 23], however is also able to capture rich contextual data surrounding eating occasions. The wide limits of agreement seen in the Bland-Altman plot suggest this method would be more suitable for dietary assessment at a group level rather than individual level.

The results presented within this study are consistent with previous validation studies evaluating dietary assessment Smartphone applications. Twenty-four hour-recalls have been used as a reference method for two novel dietary assessment methods, namely My Meal Mate (MMM) [8] and Electronic dietary intake assessment (e-DIA) [24]. Results from both studies reported a mean EI comparable to 24-h dietary recalls, with Bland-Altman plots showing minimal bias but with wide limits of agreement [8, 24]. However, neither study assessed misreporting or accounted for under or over reporting of dietary intake.

Doubly-labelled water has also been used as a reference method for novel dietary assessment methods utilising Smartphone technology. Methods such as the Remote Food Photography Method (RFPM) [6], Nutricam Dietary Assessment Method (NuDAM) [21] and Tool for Energy Balance in Children (TECH) [22] have all assessed their respective methods for EI against DLW. When comparing EI compared to EE captured by DLW across methods; NuDAM under-reported EI, TECH adequately reported EI and RFPM documented both under-reporting and adequate-reporting depending on the kind of participant prompts used throughout the study. The RFPM method using customised prompts (participant or meal specific reminder messages) provided a comparable EI compared to those using standard prompts (general reminder message) when comparing EI to EE captured by DLW. Each method discussed was assessed in different populations and used varying data collection methodologies. For example, NuDAM was assessed in Type 2 diabetic adults and used both images and an accompanying follow-up phone call [21], TECH was assessed in children (mean age 5.5 years) and their parents, with dietary assessment based solely on the images taken by the parents of the children’s food intake [22] and RFPM was assessed in obese adults 18–54 years and relied solely on portion size estimations from participant captured images [6]. All three methods (RFPM, NuDAM and TECH) used images as an element of dietary assessment data collection. The variation in study samples and EI collection methods (e.g. images exclusively or in combination) makes comparisons between studies difficult. A recent review of Smartphone dietary assessment methods, reported Smartphone dietary assessment methods to have similar but not superior, validity or reliability when compared with conventional methods of dietary assessment [5].

The use of Smartphones as a dietary assessment platform may have a number of advantages with the potential to reduce participant burden being the most important. For example, a review of Smartphone platforms saw a reduction in the time taken to report eating occasions [17, 26] with evidence suggesting reporting of dietary intakes over longer study periods could be possible with Smartphone platforms [17, 21, 26]. In addition, costs to researchers may be reduced through the use of automated real-time coding, in which image analysis and volume estimation data can be indexed against existing nutrient databases [44–46]. This process relies on several steps, however put simply the food item needs to be identified and portion sized before being matched to an appropriate food code found in a composition database. More work is needed to improve the accuracy and consistency of these methods, with previous studies reporting problems with the illumination and angle of the images [47], difficulties in detecting hidden ingredients, cooking method, colour and texture [48] and multiple food items or mixed dishes increasing misreporting [49]. Automated coding was not implemented in the current study given the initial infrastructure costs required in establishing this process.

Participant satisfaction and preference for mobile phone dietary assessment platforms compared to conventional platforms has been highlighted in recent literature [5]. As the use of Smartphone platforms expands rapidly within the dietary assessment area, so do the number of methods which are created and not validated or evaluated. New methods require adequate evaluation in specific populations or environments to ensure they are measuring both food and nutrient intakes accurately. Without thorough evaluation, there is a risk that bias and error is not adequately considered and inappropriate conclusions are drawn from the dietary intake influencing future public health messages and interventions for fear of EI or nutrient misreporting [42].

Under and over-reporting of nutrients and EI is common within dietary assessment research [4]. Previous studies have found significant associations between under-reporting and over-reporting of food consumption. Johnson et al. [50] compared a food diary with DLW in an older population and found women were significantly more likely to under-report EI than men (*p* < 0.01), while women with higher BMIs were more likely to under-report EI than those who were of a normal BMI [50]. Similar results were seen in a study using a ratio of EI to basal metabolic rate in two 24-h recalls of adults [51]. Similar to Johnson et al. [50], Fereidoun Azizi et al. [51], found women were more likely to under-report EI than males (*p* < 0.05). They also found that UR were older and had lower BMI’s than AR (*p* < 0.01), while OR were younger, had higher BMI’s and were more likely to smoke than AR (*P* < 0.01) [51]. Both studies found misreporting related to sex and BMI while Fereidoun Azizi (2005), identified age and smoking habits also [50, 51]. These studies differed in sample ages, EI collection method, definitions for UR, OR, and AR, and approaches for establishing cut-offs [50, 51], thus making comparisons difficult. The present study did not find similar misreporting relationships, with no significant differences between ineligible reporters, UR, OR or AR for a range of participant characteristics (age, sex, BMI, country of birth, SEIFA, highest educational qualification or smoking status).

### Strengths and limitations

This study had several strengths. Firstly, MEE was objectively measured using the SWA, which has been shown to be highly accurate compared to DLW, has low participant burden, no recall bias and high compliance rates [29]. Secondly, the collection of dietary data from four non-consecutive days (including 3 week days and 1 weekend day) controlled for day-to-day variation and occasionally consumed foods [1]. This reporting period is seen as a strength compared to previous studies who reported food intake on consecutive days (5–7 days) [8, 23] placing increased burden on participants and increasing reporting error [4]. Thirdly, participants were able to download the FoodNow app on their own phones from either the Android or IPhone platforms, in comparison to previous studies where they were required to use a provided “study” device [17, 26].

Limitations of this study include the wide limits of agreement seen within the Bland-Altman Plot indicating error at an individual level. The wide levels of agreement seen within this study are consistent with previous studies which have compared electronic devices with references methods [8, 23] and suggest this form of dietary tool for dietary analysis at the population level rather than the individual level. Secondly, the sample was not representative of all young adults as 70% of participants were tertiary educated and largely female. This is a limitation as digital and computer literacy has previously been reported to be increased in those with higher socioeconomic backgrounds [52]. However, no significant differences between ineligible reporters, UR, OR or AR were reported according to age, sex, BMI, country of birth, SEIFA, highest education level or smoking status.

Thirdly, while this study was able to evaluate the ability of the FoodNow to report food and beverage intakes, other data collected by the app (contextual factors of eating occasions) were not able to be evaluated using SWA. Other Smartphone apps have also incorporated these types of measures but they have rarely been evaluated [53]. These assessments require further examination with alternative approaches such as direct observation [54] or other technology such as wearable cameras [7]. The assessment and agreement of EEI to an objective measure of MEE is the first step in substantiating that this method is a suitable and reliable method for collecting dietary intake, both nutritional and contextual. Finally, collection of images associated with the eating occasions varied between participants although it is not clear how this compares to other studies, given the lack of comparable data in the literature.

### Future directions

Future research should examine the use of FoodNow in other population groups including older age groups and those with lower education and literacy levels. Current work in this domain has found promising results with older adults engaging in Smartphone application use [55], as well as those with lower education levels [56]. Further work needs to examine alternative ways of decreasing misreporting and capturing dietary assessment as accurately as possible whilst maintaining or decreasing participant burden [42]. Future work is also required to further understand which aspects of technology are particularly advantageous in improving the accuracy of dietary assessment. For example, understanding the impact of the addition of images on the accuracy of dietary coding and comparisons between participants who reported images for every eating occasions versus those who reported images less frequently may provide further insight.

## Conclusion

Dietary assessment methods based on new technology offer great potential to improve reporting of dietary intake. However they require adequate evaluation across populations and environments to ensure they are measuring food intakes accurately. The Smartphone application FoodNow was able to accurately assess EEI when compared to MEE from SWA in young adults. Despite wide levels of agreement at the individual level (−3709 kJ to 2056 kJ), at the group level, FoodNow appears to have potential as a dietary assessment tool. Additional research is needed to confirm the validity of the FoodNow app in different populations.

## Abbreviations

AR: Adequate-reporter
BMI: Body mass index
DLW: Doubly labelled water
EE: Energy expenditure
EEI: Estimated energy intake
ICC: Intra-class correlation coefficient
kJ: Kilojoule
MEE: Measured energy expenditure
OR: Over-reporter
SEIFA: Socio-economic Index for Areas
SWA: SenseWear armband
UR: Under-reporter
